# Structures of Astaxanthin and Their Consequences for Therapeutic Application

**DOI:** 10.1155/2020/2156582

**Published:** 2020-07-20

**Authors:** Tatas Hardo Panintingjati Brotosudarmo, Leenawaty Limantara, Edi Setiyono, Heriyanto

**Affiliations:** ^1^Ma Chung Research Center for Photosynthetic Pigments (MRCPP) and Department of Chemistry, Universitas Ma Chung, Villa Puncak Tidar N01, Malang 465151, Indonesia; ^2^Center for Urban Studies, Universitas Pembangunan Jaya, Jl. Cendrawasih Raya B7/P, South Tangerang, 15413 Banten, Indonesia

## Abstract

Reactive oxygen species (ROS) are continuously generated as a by-product of normal aerobic metabolism. Elevated ROS formation leads to potential damage of biological structures and is implicated in various diseases. Astaxanthin, a xanthophyll carotenoid, is a secondary metabolite responsible for the red-orange color of a number of marine animals and microorganisms. There is mounting evidence that astaxanthin has powerful antioxidant, anti-inflammatory, and antiapoptotic activities. Hence, its consumption can result in various health benefits, with potential for therapeutic application. Astaxanthin contains both a hydroxyl and a keto group, and this unique structure plays important roles in neutralizing ROS. The molecule quenches harmful singlet oxygen, scavenges peroxyl and hydroxyl radicals and converts them into more stable compounds, prevents the formation of free radicals, and inhibits the autoxidation chain reaction. It also acts as a metal chelator and converts metal prooxidants into harmless molecules. However, like many other carotenoids, astaxanthin is affected by the environmental conditions, e.g., pH, heat, or exposure to light. It is hence susceptible to structural modification, i.e., via isomerization, aggregation, or esterification, which alters its physiochemical properties. Here, we provide a concise overview of the distribution of astaxanthin in tissues, and astaxanthin structures, and their role in tackling singlet oxygen and free radicals. We highlight the effect of structural modification of astaxanthin molecules on the bioavailability and biological activity. These studies suggested that astaxanthin would be a promising dietary supplement for health applications.

## 1. Introduction

Carotenoids are a class of bioactive natural products synthesized by plants and certain photosynthetic microorganisms. Many carotenoids are directly involved in the photosynthesis process, while others protect the host from photooxidation and related damage [1]. Astaxanthin is a carotenoid that has garnered significant interest in recent years. It is a red xanthophyll carotenoid found predominantly in marine microorganisms and animals [2, 3]. Astaxanthin is ranked as the second most important carotenoid on the global market after capsanthin. Its market size exceeded US$ 288.7 million in 2017 and will reach US$ 426.9 million by 2022, dominating the carotenoid industry, with a predicted 8.1% compound annual growth rate until 2022 [4]. Natural astaxanthin has been approved as generally recognized as safe (GRAS). It can be sold as a dietary supplement and has been also approved as a food colorant (E161j) by the European Commission for use in the food and beverage industries [5].

Astaxanthin supplementation has been investigated in a broad range of clinical applications in humans and was shown to exert multiple pharmacological effects [3, 6]. The beneficial effects of astaxanthin are often associated with its antioxidative [7–9], anti-inflammatory [10–13], and antiapoptotic properties [10, 14–17]. As demonstrated in several studies, astaxanthin stimulates the immune response, e.g., by increasing interferon and interleukin 2 production without inducing cytotoxicity [18–21]. It potentially plays a neuroprotective role in neurological disorders, such as brain ischemic or traumatic injury, and subarachnoid bleeding [22–28]. It also exhibits a novel cardioprotective potential suppressing homocysteine-induced cardiotoxicity by alleviating mitochondrial dysfunction and oxidative damage [29]. Furthermore, astaxanthin effectively suppresses metastasis of the colon and cell lung cancers [30–32], prevents binding of the human papillomavirus L1 protein to the human sperm membrane [33], improves stem cell potency by increasing the proliferation of neural progenitor cells [34]; prevents liver damage in carbon tetrachloride-induced toxicity [35], inhibits membrane peroxidation in human endothelial cells [36], and exerts antiaging effects [37–40]. Some *in vitro* and *in vivo* studies of the biological role of astaxanthin are presented in Table 1.

Reactive oxygen species (ROS) have attracted attention as novel signal mediators involved in the modulation of cell survival, cell death, differentiation, cell signaling, and inflammation-related factor production [42]. Large quantities of ROS are generated during mitochondrial oxidative metabolism, as well as during cellular response to xenobiotics, cytokines, and bacterial invasion [43, 44]. Cellular imbalance caused by ROS or oxidants that exceed the cellular capability to mount an effective antioxidant response is called oxidative stress. It results in macromolecular damage and is implicated in various diseases. The key function of astaxanthin as a potent antioxidant depends on its ability to scavenge singlet oxygen [45] and free radicals [46].

The ability of astaxanthin to attack active oxygen species is reportedly 10-fold higher than that of zeaxanthin, lutein, tunaxanthin, cathaxanthin, and *β*-carotene, and 100-fold higher than that of *α*-tocopherol [47]. The unique molecular structure of astaxanthin, the characteristics of its excited state and isomeric forms, and the tendency to aggregate in different solvents impact its biological activity and bioavailability. In the present review, we provide information on astaxanthin sources, structural diversity, and mechanism of action related to its interaction with ROS.

## 2. Sources of Astaxanthin

The natural sources of astaxanthin include green algae, bacteria, fungi, archaea, chromista, shrimp, crawfish, crabs, lobster, Antarctic krill, marine copepoda, and salmonids, as presented in Table 2.

Astaxanthin is ubiquitous in marine organisms. It is responsible for the well-known red-orange color of the skin and flesh of shrimp and crayfish, and the flesh of salmon. Crustaceans and fish cannot synthesize astaxanthin *de novo*, and thereby rely on the supply of astaxanthin precursors through the consumption of algae and other microorganisms [87]. Likewise, human is unable to synthesize carotenoids, and they have to be obtained from the diet. The main source of astaxanthin for humans is seafood, with salmon, for example, wild Sockeye salmon (*Oncorhynchus nerka*), containing the most astaxanthin (26–38 mg kg^–1^ wet weight) [86]. The rainbow trout (*Oncorhynchus mykiss*) is also a good source of astaxanthin (24 mg kg^–1^ wet weight) [86]. Currently, approximately 72% of the world's salmon production is farmed [88]. Flesh pigmentation is a key commercial trait of farmed salmon, and it is often associated with product quality. In the aquaculture sector, commercial astaxanthin food additives can account for up to 25% of feed costs [89, 90]. The origin of astaxanthin sources can be monitored by high-performance liquid chromatography (HPLC) of the isomers present in the flesh. For instance, in salmon fed yeast-derived astaxanthin, the 3*R*,3′*R* isomer is the major component, while in salmon fed synthetic astaxanthin, the 3*R*,3′*S* isomer is abundant [91]. Of note, currently, over 95% of astaxanthin available on the market is derived synthetically from petrochemicals; on the other hand, *Haematococcus pluvialis*-derived natural astaxanthin accounts for <1% of commercialized astaxanthin [92]. However, safety concerns have arisen regarding the use of synthetic astaxanthin for human consumption [93].

## 3. Astaxanthin Structures

### 3.1. Basic Structure and Isomers of Astaxanthin

**Figure 1:**
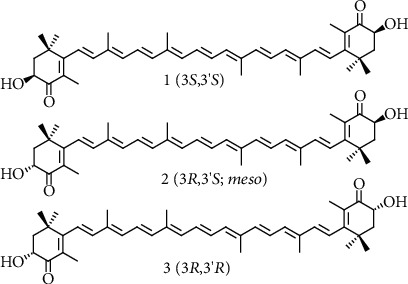
Structures of the optical isomers all-*E*-(3*S*,3′*S*) (1), all-*E*-(3*R*,3′*S*; *meso*) (2), and all-*E*-(3*R*,3′*R*) (3) astaxanthin.

Astaxanthin is a xanthophyll carotenoid because it contains oxygen. Astaxanthin (3,3′-dihydroxy-*β*,*β*′-carotene-4,4′-dione; CAS no. 472-61-7; Mw = 596.8 g mol^–1^; *ε*_468 nm_ = 125 × 10^3^ mol^–1^ cm^–1^ in hexane) consists of two terminal *β*-ionone–type rings joined by a polyene chain. It has two asymmetric carbons located at the 3,3′-position of the *β*-ionone ring, with a hydroxyl group (-OH) on either end of the molecule (Figure 1). Oxygen is present in the ring system as both a hydroxyl and a keto (C=O) group.

Astaxanthin can be esterified, which increases its solubility in the cell and makes it more stable to oxidation [94]. The hydroxy group on one or both rings can bind to different fatty acids, such as palmitic, oleic, estearic, or linoleic acid, to form mono- or diesters, accordingly. Astaxanthin also exists in a free form, i.e., with the hydroxyl group not esterified, and in a chemical complex with proteins or lipoprotein. Further, stereoisomers and geometric isomers of astaxanthin are identified [81]. Figure 1 shows three configurational isomers of the compound: two enantiomers (3*R*,3′*R* and 3*S*,3′*S*) and a *meso* form (3*R*,3′*S*). The alga *Haematococcus* synthesizes mainly the 3*S*,3′*S* isomer, which is also predominant in wild Atlantic salmon, and occurs mainly in the free form [93]. The Antarctic krill (*Euphausia superba*) produces the 3*R*,3′*R* as the primary isomer [3]. Whereas the three optical stereoisomers exist in nature in variable ratios, synthetic astaxanthin is a racemic mixture of the two enantiomers and the *meso* form [81]. Synthetic astaxanthin contains the 3*S*,3′*S*, 3*R*,3′*S*, and 3*R*,3′*R* isomers in a 1 : 2 : 1 ratio, respectively. It is not esterified, while natural astaxanthin mostly occurs in esterified form, or in a complex with proteins or lipids [81].

**Figure 2:**
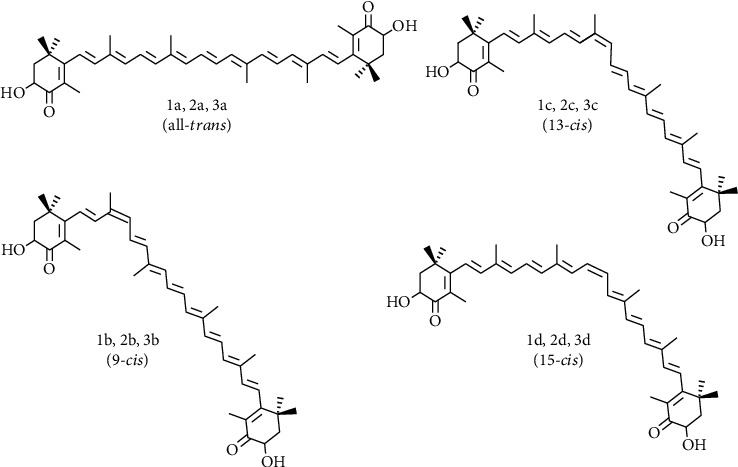
Structure of all-*trans* (1a, 2a, 3a), 9-*cis* (1a, 2b, 3c), 13-*cis* (1c, 2c, 3c), and 15-*cis*-astaxanthin (1d, 2d, 3d). Note: 1, 3*S*,3′*S*; 2, 3*R*,3′*S*; *meso*; 3, 3*R*,3′*R*.

Astaxanthin also exists as *trans* and *cis* (*E* and *Z*) geometrical isomers (Figure 2), depending on the configuration of the double bonds in the polyene chain. All-*trans* astaxanthin (all-*E*-astaxanthin) is the dominant isomer, although at least two *cis*-isomers (9-*cis* and 13-*cis*) also occur in nature, depending on the host species and body part, and are also found in synthetic preparations [95]. In rainbow trout (*O. mykiss*), most astaxanthin is present as all-*trans* astaxanthin (97%), followed by 9-*cis* (0.4%), 13-*cis* (1.5%), and other isomers (1.1%) [95, 96]. Further, 15*-cis* and di-*cis* isomers were identified in addition to all-*trans*, 13-*cis*, and 9-*cis* isomers, in various wild and cultured shrimps in China, *Trachysalambria curvirostris*, *Penaeus monodon*, *Fenneropenaeus chinensis*, *Litopenaeus vannamei*, and *Exopalaemon carinicauda* [97, 98].

The isomerization of *trans*-astaxanthin to *cis*-isomers has been investigated in various organic solvents [99]. All-*trans* natural astaxanthin is readily isomerized to *cis*-*trans* mixtures, especially the 9-*cis* and 13-*cis* isomers. Increased temperature, exposure to light, or the presence of acids can result in the formation of *cis*-isomer [99, 100]. Isomerization of all-*trans* astaxanthin is challenging because of low stereochemical stability, as well as other factors, such as microwave radiation, ultrasonic vibration, organic solvents, presence of fatty acids, and Cu (II) ion, which affect the formation yield of different astaxanthin isomers [99–103]. Therefore, isomerization of *trans-*astaxanthin and *cis*-isomers has to be minimized during the preparation of pure isomeric forms, i.e., during pigment extraction, astaxanthin ester saponification, and isomer purification. Reversed-phase HPLC can be used to separate the *trans* and *cis*-isomers of astaxanthin [100].

### 3.2. Structure of Astaxanthin Aggregates

Biomolecular aggregation is important in medical treatments. Like many other carotenoids, astaxanthin is expected to self-assemble in hydrated polar solvents to form aggregates [104]. Water concentration in these mixtures impacts the aggregate morphology and greatly affects their photophysical properties. Based on the spectroscopic analysis, the aggregate absorption spectra are blue-shifted or red-shifted compared with those of monomers, depending on the aggregation conditions [105–107]. Specifically, the red-shift is attributed to the formation of *J*-aggregate and the blue-shift is attributed to the formation of *H*-aggregate. *J*-aggregate consists of astaxanthin molecules arranged head-to-tail and forming a relatively loosely packed aggregate, while *H*-aggregate is characterized by tight “card-pack” stacking where the polyene chains are more or less parallel to each other [108].

Aggregation of astaxanthin in ethanol–water solution has been studied by ultraviolet/visible and fluorescence spectrometry. After the addition of water, astaxanthin immediately forms tightly packed stacks of individual molecules, with a maximum blue shift of 31 nm; the *J*-aggregate forms in 1 : 3 (*v*/*v*) ethanol–water solution after an hour [107]. In the case of dimethyl sulfoxide (DMSO), an organic solvent that is usually used for delivering carotenoid in cell culture, the addition of water for a ratio of 1 : 1 (*v*/*v*), generates a red-shifted *J*-aggregate with a maximum absorption band at 570 nm. On the other hand, the DMSO/water ratio of 1 : 9 (*v*/*v*) produces blue-shifted *H*-aggregate with maximum absorption bands at 386 nm and 460 nm [105]. Of note, the formation of astaxanthin aggregates also changes the excited-state dynamic of the compound, i.e., in DMSO/water solution, it results in a longer S_1_ lifetime than that of the corresponding monomer, with the astaxanthin triplet generated exclusively in the astaxanthin aggregate [105]. In nature, astaxanthin also forms monoesters and diesters with fatty acids. Ester formation completely prevents aggregation [109].

### 3.3. Structure and Bioavailability

As mentioned above, bioavailability of astaxanthin in aquatic animals has been well researched. The all-*trans* isomer is predominantly found in most body parts of the aquatic animals, including fish and crustaceans. The 13-*cis* isomer is present in the fish liver at relatively high amounts [95, 96]. In other words, the configuration of the astaxanthin molecule may influence its absorption, as reported for rainbow trout fed cold-pelleted diets containing select astaxanthin isomers [96]. This resulted in the formulation of the following hypotheses [57]: (1) an isomer that is easily ingested and absorbed accumulates faster than other isomers in an aquatic animal; (2) the isomer is selectively transported in the plasma and between tissues or organs; and (3) astaxanthin may undergo metabolic conversion before deposition in the animal. The higher absorption of all-*trans* astaxanthin than that of the *cis*-isomer may be explained by the relatively lower ability of the sterically bulkier *cis-*isomer to permeate the lipid membrane [110].

Bioavailability of natural astaxanthin stereoisomers from wild and farmed salmon in healthy men was assessed in a randomized and double-blind trial involving 28 volunteers [111]. The participants were given 250 g of wild or aquaculture salmon (5 *μ*g g^−1^) daily, for 4 weeks. The plasma astaxanthin concentration and isomer distribution were monitored by HPLC. On day 28, the 3*S*,3′*S* isomer was predominant in the plasma (80%) of individuals in the wild salmon intake group, whereas the *meso* form (3*R*,3′*S*) was prevalent (48%) in the farmed salmon intake group. Although the all-*trans* astaxanthin has been the subject of many studies, according to recent research, the 9-*cis* and 13-*cis* isomers are selectively absorbed by human plasma. Bioavailability of synthetic astaxanthin was also assessed in the plasma and lipoprotein fractions in three middle-aged male volunteers after ingestion of a single meal containing a 100 mg dose of astaxanthin mixture of all-*trans* (74%), 9-*cis* (9%), and 13-*cis* isomers (17%) [110]. Astaxanthin was present mainly in very low-density lipoproteins containing chylomicrons (VLDL/CM; 36-64%), whereas low-density lipoprotein (LDL) and high-density lipoprotein (HDL) contained 29% and 24% of total astaxanthin, respectively. The astaxanthin isomers in HDL, LDL, and VLDL/CM ranged from 62.1–66.9% for all-*trans* isomer, 13.2–14.5% for 9-*cis* isomer, and 20.4–24.3% for 13-*cis* isomer, respectively.

Recently, bioaccessibility and bioavailability of all-*trans*, 9-*cis*, and 13-*cis* astaxanthins was investigated using an *in vitro* digestion model and human intestinal Caco-2 cells [112]. Indeed, the 13-*cis* astaxanthin was more bioaccessible than the 9-*cis* and all-*trans* forms during *in vitro* digestion, and 9-*cis* astaxanthin was transported in human intestinal Caco-2 cells more efficiently than the all-*trans* and 13-*cis* isomers [112]. In terms of absorption efficiency of all isomers during transport across differentiated Caco-2 cell monolayers, cellular uptake was pronounced after 3 h, and the absorption efficiency increased drastically after 12 h and continued to increase until the 24 h time point. The absorption efficiency of all-*trans* isomer was the highest, followed by those of 9-*cis* and 13-*cis* isomers, indicating that the linear structure of the isomer initially facilitates its permeation of the cell membrane, as compared with the sterically bulky *cis*-isomers. In the experiment, *cis*-astaxanthin was more readily isomerized to all-*trans* isomer than to another *cis*-isomer, although all-*trans* astaxanthin tended to be isomerized to 13-*cis* astaxanthin slightly more readily than to 9-*cis* astaxanthin [112]. In the experiment, DMSO was used to deliver astaxanthin to the cultured cells. The observations highlight the possible effect of structure on the biological role of these isomers, especially in cellular uptake and transport.

## 4. Interaction of Astaxanthin with Reactive Species

Harmful effects of reactive species are well established. As determined in studies involving animal models and human subjects, reactive species participate in the pathogenesis of acute and chronic diseases. That is because nucleic acids (RNA and DNA), and proteins are the main targets of free radicals [113, 114].

Inflammation widely contributes to multiple chronic diseases and is closely linked with oxidative stress. For example, elevated levels of prooxidants and various markers of oxidative stress, and cell and tissue damage are linked to the pathogenesis of cancer, cardiovascular disease, neurodegenerative diseases, reproductive system diseases, and aging [115]. Accumulation of an antioxidant in a cell can reduce or prevent oxidation of oxidizable substrates. An ideal antioxidant should be readily absorbed, eliminate free radicals, and chelate redox metals at physiologically relevant levels [116].

Free radicals contain one or more unpaired electrons. This feature makes them particularly reactive and is also responsible for their ability to trigger chain reactions, propagating the associated molecular damage. Most free radicals are, or arise from, reactive oxygen species (ROS), reactive nitrogen species (RNS), and reactive sulfur species (RSS). ROS include oxygen-based free radicals, i.e., the superoxide radical anion (O_2_^•–^), and hydroxyl (HO^•^), alkoxyl (RO^•^), organic peroxyl (ROO^•^), and hydroperoxyl (HOO^•^) radicals. RNS comprise peroxynitrite (ONOO^–^), nitric oxide (NO^•^), and nitrogen dioxide (NO_2_^•^). The most common RSS are thiyl radicals (RS^•^), sulfenic acids (RSOH), and disulfide-S-oxides [RS(O)_2_SR]. Concerning reactivity, HO^•^ is the most reactive and dangerous species among ROS. ROO^•^ is significantly less reactive than HO^•^, which allows them to diffuse to remote cellular locations [117]. For RNS, the chemical reactivity of NO^•^ is low, but it reacts with O_2_^•–^ yielding peroxynitrite, a highly damaging species, to react with lipids, protein, and DNA [117].

Since RNS and RSS are less reactive than the reactive oxygen species, below, we focus on ROS.

### 4.1. Generation of ROS

In the cell, ROS are produced by enzymes of different origin, mainly by the cytoplasmic membrane NADPH oxidase; the enzyme complex of the mitochondrial respiratory chain; and such enzymes as xanthine oxidase, lipo- and cyclooxygenase, and cytochromes P450 in the endoplasmic reticulum, peroxisomes, and others [43]. The mitochondrial respiratory chain is a main contributor, as 85% of oxygen metabolized the mitochondrion and partially reduced oxygen intermediates are produced therein [118]. In this organelle, O_2_^•–^ and H_2_O_2_ participate in redox signaling [119], but their production is greatly enhanced during oxidative stress, for example in response to various diseases or stimuli.

**Figure 3:**
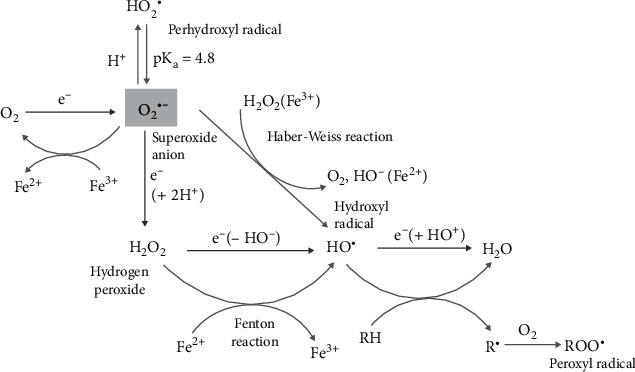
Chemical players in the generation of reactive oxygen species (ROS): superoxide anion (O_2_^•–^) radical, perhydroxyl (HO_2_^•^) radical, metal-catalyzed conversion of superoxide anion (O_2_^•–^) and hydrogen peroxide (H_2_O_2_) into hydroxyl (HO^•^) radical, and peroxyl radical (ROO^•^).

Oxidative stress reflects an imbalance between the production of ROS and the action of the antioxidant defense system in charge of their neutralization. The defense system includes superoxide dismutase, which reduces O_2_^•–^ to H_2_O_2_ [120], and catalase, glutathione peroxidase, and thioredoxin reductase, which regulate H_2_O_2_ levels by converting it to H_2_O and O_2_ [121, 122]. The production of HO^•^ was usually obtained by Fenton reaction, with Fe^2+^ reducing H_2_O_2_ to HO^•^ and HO^–^ (Figure 3). In this case, ROS are generated by a metal-catalyzed reaction and the resulting oxidative damage is often site-directed, particularly when the biomolecules coordinate metal ions [123].

In a biological context, the toxicity of O_2_^•–^ is indirect since the species is involved in the generation of highly reactive secondary species (HO^•^) [124]. The latter is generated by the reaction of superoxide (O_2_^•–^) and hydrogen peroxide (H_2_O_2_) (the Haber-Weiss reaction) (Figure 3). The reaction is thermodynamically, but not kinetically, favorable, and has to be catalyzed by a metal ion, for example, ferrous iron [125].

Further, H_2_O as an oxidant is not thermodynamically favorable under biological conditions [E°(O_2_^•–^/H_2_O_2_) = 0.93 V and E°(H_2_O_2_/HO^•^) = 0.30 V] [126]. The highest oxidizing potential of this molecule is achieved indirectly, when it is converted into HO^•^ radicals via the metal-catalyzed Fenton and Haber-Weiss reactions (Figure 3).

The hydroxyl radical is a powerful oxidant among ROS, with a potential of E°(HO^•^/H_2_O) = 2.34 V [126]. It can be converted to a relatively less reactive oxide radical, O^•–^, at very low pH [pK_a_(HO^•^/O^•–^) = 11.9] [127]; however, this conversion is not relevant at physiological pH.

The HO^•^ radical is electrophilic and has a strong affinity for electron-rich sites of molecules [127]. The radical targets numerous and various biomolecules via H-abstraction, leading to the formation of carbon-centered radicals that rapidly react with molecular oxygen to generate the peroxyl radical (ROO^•^). The latter reacts faster than the superoxide anion with numerous biological substrates (DNA, lipids, and proteins) with the rate constant ranging from 10^2^ to 10^8^ L mol^–1^ s^–1^ [128].

### 4.2. Astaxanthin Structure and Antioxidant Activity

A qualified antioxidant should be involved in one or more of the following processes to protect a biological system against oxidative damage [2]: (i) oxygen depletion; (ii) quenching of singlet oxygen molecules; (iii) scavenging of ROS or termination of a chain reaction of oxidation propagation; (iv) chelation of metal ion that otherwise could catalyze ROS formation; or (v) repair of oxidative damage. A range of ROS is found in the human body. Accordingly, the essential role of astaxanthin as an antioxidant is to deactivate reactive oxidants, such as singlet oxygen and ROS (processes 1-3), in particular, peroxyl radical intermediates [47, 80, 129].

The scavenging reaction not only depends on the carotenoid structure but also on the properties of free radicals [130]. Indeed, certain radicals elicit electron transfer, while others only engage in an addition reaction [131]. The environment plays an important role as well, with the solvent polarity leading to different mechanisms of radical–carotenoid interactions [131]. In general, three possible mechanisms of carotenoid (Car in the equations below) interactions with ROS exist: (i) hydrogen abstraction or hydrogen atom transfer from the side chain, i.e., C4-position of the cyclohexene ring (Equation (1)); (ii) radical addition to the polyene chain (Equation (2)); and (iii) electron transfer yielding a carotenoid radical cation (Equation (3)) [132, 133].

(1) $$\begin{array}{c} {\text{ROO}}^{•}+\text{Car}\to \text{ROOH}+{\text{Car}}^{•}, \end{array}$$

(2) $$\begin{array}{c} {\text{ROO}}^{•}\;+\text{Car}\to {\left(\text{ROO}–\text{Car}\right)}^{•}, \end{array}$$

(3) $$\begin{array}{c} {\text{ROO}}^{•}+\text{Car}\to {\text{ROO}}^{—}+{\text{Car}}^{•+}, \end{array}$$

(4) O12∗+Car→O32+C3ar∗

Many well-documented examples of reaction mechanisms corresponding to the reactions described by Equations (2) and (3) are known; however, the majority concern *β*-carotene [132–135]. Astaxanthin is a carotenoid with the best scavenging capacity for peroxyl and hydroxyl radicals [136, 137]. The HO^•^ scavenging capacity of astaxanthin is 66% and 17% greater than that of Trolox and quercetin, respectively, and only 6% less potent than that of *α*-tocopherol [136]. However, with respect to ROO^•^, astaxanthin is 16% more potent than *α*-tocopherol [136]. Hypothetically, the keto group potentially activates the hydroxyl group, resulting in the formation of an ortho-dihydroxy–conjugate polyene system that facilitates hydrogen transfer (Equation (1)) to the peroxyl radical, in a manner similar to that of the hydroxyl group of *α*-tocopherol [138]. As determined in an electrochemical study of carotenoid in solution using cyclic voltammetry, upon electron transfer from the carotenoid molecule, the radical cation Car^•+^ is formed at an oxidation potential E_1_°. The second lost electron forms the dication Car^2+^ at an oxidation potential E_2_°. The electrochemical measurement of astaxanthin, and astaxanthin monoester and diester, indicated that astaxanthin [E_1_°(Car/Car^•+^) = 0.768 V] has a higher oxidation potential than other carotenoids, such as *β*-carotene [E_1_°(Car/Car^•+^) = 0.634 V], zeaxanthin [E_1_°(Car/Car^•+^) = 0.63 V], and lycopene [E_1_°(Car/Car^•+^) = 0.60 V] [135, 139]. Furthermore, electrochemical in combination with electron paramagnetic resonance (EPR) spin trapping studies demonstrated that with an increasing first oxidation potential of the carotenoid molecule, the relative scavenging ability also increases, towards peroxyl radicals HOO^•^ formed in a Fenton reaction via the reaction between H_2_O_2_ and HO^•^ [139, 140]. The antioxidative effects of astaxanthin were evaluated in 35 individuals who underwent bilateral cataract surgery by monitoring the changes in superoxide scavenging activity, and hydrogen peroxide and total hydroperoxide levels in human aqueous humor [141]. After astaxanthin intake, the superoxide scavenging activity was greatly elevated, while the total hydroperoxide levels were significantly lowered [141].

Based on *in vitro* studies, astaxanthin esters act as powerful quenchers of singlet oxygen under both hydrophilic and hydrophobic conditions [142]. Kinetic studies of the quenching reaction of singlet molecular oxygen (^1^O_2_) by carotenoids and food extracts in solution revealed that carotenoids with 11 carbon atoms involved in the *π*-conjugation (*n*), such as astaxanthin, quench ^1^O_2_ most effectively [143, 144]. The quenching mechanism was proposed to involve an energy transfer from the singlet oxygen to produce the carotenoid triplet state (^3^Car) (Equation (4)) because of the two oxo groups in the conjugated astaxanthin system [145–147]. Astaxanthin in the energy-rich triplet state can return to the ground state by dissipating the energy as heat [146]. Recent quantum dynamic calculations and *ab initio* calculations suggest that once ^1^O_2_ and carotenoids make a van der Waals contact, the strong electronic coupling induces an ultrafast ^1^O_2_ quenching, which overcomes the large energy gap to the Car^•+^/O_2_^•–^ intermediate states [148]. Effects of astaxanthin supplementation on aging have been examined in the model organism *Caenorhabditis elegans*. Indeed, astaxanthin effectively quenched ^1^O_2_, thus protecting the mitochondrion and nucleus from oxidative injury and extending the lifespan [149].

The antioxidant capacity of astaxanthin can be determined from the quenching rate constant of singlet oxygen. A fast quenching rate constant indicates efficient singlet oxygen quenching. The quenching rate constants of singlet oxygen for astaxanthin determined by various methods and in various environments are shown in Table 3. The rate constants of astaxanthin in organic solvents (18–240 × 10^8^ M^–1^ s^–1^) are higher than those in micelle (71.1 × 10^8^ M^–1^ s^–1^) and liposome (0.19–5.9 × 10^8^ M^–1^ s^–1^). The rate constants in heterogeneous environment are lower than those in organic solvents, which can be explained by a slower diffusion of singlet oxygen from the aqueous phase to the micelle or lipid membrane in these environments [150]. In addition, the quenching rate constants of astaxanthin using Rose Bengal- and 12-(1-pyrene)-dodecanoic acid-sensitized photooxidation methods are 100–667-fold (in ethanol) and 1–8-fold (liposomes) higher than those of *α*-tocopherol [151].

**Figure 4:**
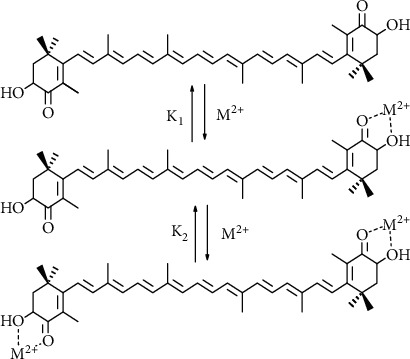
Complexation of astaxanthin with metal ions (adaptatrd from [161]).

The molecular structure of astaxanthin endows it with unique properties. Hydroxyl and keto groups are present at positions C3 (C3′) and C4 (C4′), respectively, on each ionone ring. The two adjacent oxygen atoms on the cyclohexene ring enable the formation of stable complexes with metal ions (Figure 4), as is also observed in many *α*-hydroxyketones and hydroxyquinones [157, 158]. The generation of highly reactive oxygen species (HO^•^) via Fenton and photo-Fenton reactions *in vitro* can be completely inhibited by the chelation of metal ions Fe^2+^ or Cu^2+^ [159]. According to a density functional theory study, astaxanthin may form metal ion complexes with such metal ions as Ca^2+^, Cu^2+^, Pb^2+^, Zn^2+^, Cd^2+^, and Hg^2+^ [160]. Indeed, the interaction of astaxanthin with metal ion complexes, i.e., Ca^2+^, Zn^2+^, and Fe^2+^, was evaluated by mass spectrometry and nuclear magnetic resonance. The analysis revealed that the two oxygen atoms at the terminal cyclohexene rings chelate the metal to form 1 : 1 complexes at low Ca(ClO_4_)_2_, Zn(ClO_4_)_2_, and Fe(ClO_4_)_2_ salt concentrations; at high salt concentrations (>0.2 mM), a 2 : 1 salt to astaxanthin complex is formed [161]. Furthermore, the stability constant of 1 : 1 astaxanthin complex with Fe(ClO_4_)_2_ was calculated to be K_1_ = 3000 M^–1^, whereas for 1 : 1 complex with Ca(ClO_4_)_2_, the stability constants are K_1_ = 23,000 M^–1^ and K_2_ = 5000 M^–1^. The ability to form chelate complexes with metals could be related to the efficiency with which astaxanthin acts as a protective agent, particularly in inhibiting hydroxyl radicals [135].

The geometrical isomers of astaxanthin play an important role in its antioxidant activity. A microassay evaluating the peroxyl radical scavenging capacity of 15 carotenoid standards has been developed and validated, specifically to study the structure–activity relationship [162]. The values for ROO^•^ scavenging capacity were then calculated using *α*-tocopherol as a reference compound. Among the carotenoids studied, all-*trans* astaxanthin was identified as a highly efficient ROO^•^ scavenger (6.50 ± 0.62, *α*-tocopherol relative) [162]. As determined by a radical 2,2-diphenyl-1-picrylhydrazyl (DPPH) scavenging activity test and lipid peroxidation test, the *cis* isomers of astaxanthin, especially the 9-*cis* isomer, showed a higher antioxidant potential *in vitro* than all-*trans* astaxanthin [163]. Furthermore, among all astaxanthin isomers, the 9-*cis* isomer also most effectively inhibits the generation of ROS induced by 6-hydroxydopamine in human neuroblastoma SH-SY5Y cells, as well as the degradation of collagen type II induced by docosahexaenoic acid (DHA) and linoleic acid hydroperoxides [163]. The 13-*cis* astaxanthin had higher antioxidant activity than all-*trans* and 9-*cis* in oxygen radical absorbing capacity assay for lipophilic compounds, photochemiluminescence, and cellular antioxidant activity assay [164]. It was shown that all isomers were relatively stable between pH 2.0–11.6, except for 9-*cis* and 13-*cis* astaxanthin at pH 2. Different methods of antioxidant evaluation and antioxidant activity of astaxanthin and *cis-trans* isomers are summarized in Table 4.

## 5. Conclusion

In the last decade, studies on astaxanthin as a potent therapeutic agent have shown encouraging results. Astaxanthin can potentially be used to address various human health issues, including cancer, cardiovascular and neurodegenerative diseases, and aging. These diseases are mostly associated with inflammation caused by an interaction of nucleic acids and proteins with harmful reactive species. These effects are related to the unique properties of astaxanthin molecular structure that allow it to scavenge reactive species and quench singlet oxygen. Recent experiments have uncovered the contribution of different astaxanthin isomers to antioxidant activities, both *in vitro* and *in vivo*. However, there is a lack of research on astaxanthin and its metabolism in biological systems. Future research should focus on the physicochemical properties of different astaxanthin structures, their uptake mechanisms, and the facility of incorporation into metabolic pathways. Molecular studies involving *in vitro* and *in vivo* models should also be performed to investigate the nutraceutical and pharmaceutical applications of various astaxanthin isomers.

## Figures and Tables

**Table 1:** Biological activities of astaxanthin evaluated *in vitro* and *in vivo*, with the study model, target, and dosage specified.

Model	Main effect	Dosage	Target	Disease	Ref.
SD rat	Antioxidant	10 *μ*M	Nrf2/ARE	Diabetic nephropathy	[7]
C57BL/6 mice	Antioxidant	0.02% (*w*/*w*)	Nrf2, HO-1, BALF	Chronic obstructive pulmonary melanogster	[8]
ETS mouse	Anti-inflammatory	40 and 80 mg/kg	p38 MAPK, NF-*κ*B-p65, PSD-95, IL-6, TNF-*α*, MDA, SOD, GSH, CAT	Cognitive impairment	[10]
*H. Pylori*-free BALB/c female mice	Anti-inflammatory	10 or 40 mg/d	IFN-*γ*, IL-2, IL-10	Gastrointestinal disease	[21]
*Drosophila melanogaster* (fruit fly)	Antiaging	10 or 20 mg/mL	SOD1, SOD2, CAT	No detailed disease	[39]
Wistar rat	Antiaging	10 mg/kg/d	IL-1*β*, IL-10	Brain aging	[40]
Wistar rat	Anticancer	15 mg/kg/d	ERK-2, NF-*κ*B-p65, COX-2, MMPs-2/9, AKT, ERK-2	Colon cancer	[31]
Nonsmall cell lung cancer type A549 and H1703	Anticancer	2.5–20 *μ*M	Rad51, AKT	Lung cancer	[32]
Male BALB/c mice	Immunomodulatory	0.28, 1.4, and 7 mg/kg/d	IFN-*γ*, IL-2, Con A, LPS	No detailed disease	[18]
HPV16-L1	Anti-HPV	2 *μ*M	CTB, Lyn, Tyr-P, ARC	HPV	[33]
Male SPF C57BL/J mice	Antiobesity	0.005% or 0.01%/diet powder	Weight gain, energy intake, fat index, plasma triacylglycerol and cholesterol, liver triacylglycerol and cholesterol	Obesity	[41]
Wistar rat	Neuroprotective	10 mg/kg	GPx, MDA	Alzheimer's disease	[23]
HT22 cells	Neuroprotective	1.25–5 *μ*M	HO-1, Nrf2, Bcl-2, Bax, AIF, Cyto-c, p-Akt and p-GSK-3*β*	Alzheimer's disease	[24]
Wistar rat	Neuroprotective	10 *μ*L of 0.2 mM	Bax, Bcl-2, cleaved-caspase-3	Spinal cord injury	[25]
Human cell line SH-SY5Y	Antiapoptotic	1–20 *μ*M	6-OHDA	Parkinson's disease	[15]
Balb/C mice	Antiapoptotic	20 and 40 mg/kg	TNF-*α*, Bcl-2	Autoimmune hepatitis	[17]
Neural progenitor cells	Proproliferative	1, 5, and 10 ng/mL	PI3K, MEK	Alzheimer's disease	[34]
H9c2 rat myocardial cells	Cardioprotective	0.5–8 *μ*M	Bcl-2	Cardiovascular diseases	[29]
Mouse	Cardioprotective	5 mg/kg/d	GSH-Rs and MDA	Cardiovascular diseases	[29]
Albino Wistar rat	Hepatoprotective	100 and 250 *μ*g/kg	SGPT, SGOT, ALP	No specific disease	[35]

Note: SD: Sprague-Dawley; Nrf2/ARE: nuclear factor-erythroid 2-related factor 2/antioxidant response element; HO-1: heme oxygenase-1; BALF: bronchoalveolar lavage fluid; p38 MAPK: p38mitogen-activated protein kinase; NF-*κ*B-p65: nuclear factor-kappa B-p65; PSD-95: postsynaptic density protein 95; IL-6: interleukin-6; TNF-*α*: tumor necrosis factor; MDA: malondialdehyde; SOD: superoxide dismutase; GSH: glutathione; CAT: catalase; IFN-*γ*: interferon gamma; IL-2: interleukin 2; IL-10: interleukin 10; IL-1*β*: interleukin 1 beta; ERK-2: extracellular signal-regulated kinase-2; COX-2: cyclooxygenase-2; MMPs2/9: matrixmetallo proteinases; Akt: protein kinase B; Rad51: Rad51 genes; Con A: concanavalin A; LPS: lipopolysaccharide; CTB: cholera toxin subunit B; Lyn: tyrosine kinase Lyn; ARC: acrosome-reacted cells; GPx: glutathione peroxidase enzyme; MDA: malondialdehyde; AIF: apoptosis-inducing factor; Cyto-c: cytochrome-c; GSK-3*β*: glycogen synthase kinase-3*β*; Bax: Bcl-2 associated X protein; Bcl-2: B-cell lymphoma 2; GSH-Rs: reduced glutathione; SGPT: serum glutamate transaminase; SGOT: serum glutamate oxaloacetate; ALP: alkaline phosphatase.

**Table 2:** Natural sources of astaxanthin.

Source	Example of species	Astaxanthin concentration (mg kg^−1^)^∗^	Primary optical isomer	Reference
Microorganism				
Phytoplankton	*Haematococcus pluvialis* NIES-144	98,000 *d.w.*	3*S*,3′*S*	[48]
	*Haemotococcus lacustris*	43,100 *d.w.*	3*S*,3′*S*	[49]
	*Haematococcus pluvialis* CCAP-34/7	22,700 *d.w.*	3*S*,3′*S*	[50]
	*Neochloris wimmeri* CCAP-213/4	19,200 *d.w.*	—	[50]
	*Protosiphon botryoides* SAG-731/1a	14,300 *d.w.*	—	[50]
	*Scotiellopsis oocystiformis* SAG-277/1	10,900 *d.w.*	—	[50]
	*Chlorella zofingiensis* SAG-211/14	6800 *d.w.*	—	[50]
	*Scenedesmus vacuolatus* SAG-211/15	2700 *d.w.*	—	[50]
	*Chlorococcum* sp.	4230 *d.w.*	—	[51]
	*Chromochloris zofingiensis*	13,100 *d.w.*	—	[52]
	*Euglena sanguinea*	—	3*S*,3′*S*	[53]

Zooplankton	*Calanus helgolandicus*	50–220 *d.w.*	—	[54]
	*Acartia bifilosa*	477.4 *d.w.*	—	[55]
	*Calanus finmarchicus*	100–500 *d.w.*	3*S*,3′*S*	[56, 57]
	*Calanus glacialis*	100–500 *d.w.*	—	[56]
	*Calanus hyperboreus*	100–500 *d.w.*	—	[56]
	*Calanus pacificus*	—	—	[58]
	*Diaptomus nevadensis*	—	—	[58]
	*Neocalanus tonsus*	—	—	[58]
	*Amphiascoides atopus*	619 *d.w.*	—	[59]
	*Acartia bifilosa*	293–487 *d.w.*	—	[60]
	*Pseudocalanus acuspes*	239–305 *d.w.*	—	[60]
	*Idotea metallica*	—	—	[61]

Bacteria	*Agrobacterium aurantiacum*	140 *d.w.*	3*S*,3′*S*	[62]
	*Brevundimonas* sp. strain N-5	837 *d.w.*	3*S*,3′*S*	[63]
	*Sphingomicrobium astaxanthinifaciens*	40 *d.w.*	—	[64]
	*Paracoccus bogoriensis*	400 *w.w.*	—	[65]
	*Brevundimonas* spp.	27.6–365 *d.w.*	3*S*,3′*S*	[66]
	*Brevundimonas* sp. M7	1300 *d.w.*	3*S*,3′*S*	[66]
	*Sphingomonas astaxanthinifaciens*	690 *d.w.*	—	[66]
	*Altererythrobacter ishigakiensis*	—	—	[67]
	*Paracoccus haeundaensis*	—	—	[68]
	*Paracoccus carotinifaciens*	—	3*S*,3′*S*	[69]

Yeast	*Phaffia rhodozyma* PR 190	970 *d.w.*	3*R*,3′*R*	[70]
	*Phaffia rhodozyma* UCD 67-210	387 *d.w.*	3*R*,3′*R*	[71]
	*Candida utilis*	400 *d.w.*	—	[72]

Archaea	*Halobacterium salinarium* NRC-1	265 *d.w.*	—	[73]
	*Haloarcula hispanica* ATCC 33960	17 *d.w.*	—	[73]
Chromista	*Thraustochytrium* sp. CHN-3	2800 *d.w.*	—	[74]

Crustaceans				
Shrimp	*Marsupenaeus japonicus*	418 *d.w.*	—	[75]
	*Litopenaeus setiferus*	48.3 *w.w.*	—	[76]
	*Penaeus* sp.	0.96 *w.w.* (only head)	—	[77]
	*Litopenaeus vannamei*	11.3–31.8 *w.w.*	3*R*,3′*S*	[57, 78]
	*Penaeus monodon*	24.9–67.5 *d.w.*	3*R*,3′*S*	[57, 79]
	*Pandalus borealis*	30.9–147.7 *w.w.*	3*R*,3′*S*	[57, 80, 81]

Crawfish	*Procambarus clarkii*	78.5–197.9 *d.w.* (only shell)	—	[82]
Crabs	*Pleuroncodes planipes*	—	—	[83]
	*Eriocheir sinensis*	3.5–4.7 *d.w.* (only carapace)	—	[84]
	*Chionoecetes opilio*	119.6 *d.w.*	—	[81]

Lobster	*Jasus lalandii*	13 *w.w.*	—	[85]
Antarctic krill	*Euphausia superba*	—	3*R*,3′*R*	[80]

Fishes				
Salmonids	Atlantic salmon (*Salmo salar*)	6–8 *w.w.*	3*S*,3′*S*	[3]
	Sockeye salmon (*Oncorhynchus nerka*)	26–38 *w.w.*	3*S*,3′*S*	[86]
	Chinook salmon (*Oncorhynchus tshawytscha*)	5.4 *w.w.*	3*S*,3′*S*	[86]
	Chum salmon (*Oncorhynchus keta*)	3–5 *w.w.*	3*S*,3′*S*	[86]
	Coho salmon (*Oncorhynchus kisutch*)	10–21 *w.w.*	3*S*,3′*S*	[86]
	Masu salmon (*Oncorhynchus masou*)	4.6 *w.w.*	3*S*,3′*S*	[86]
	Pink salmon (*Oncorhynchus gorbuscha*)	4–7 *w.w.*	3*S*,3′*S*	[86]
	Rainbow trout (*Oncorhynchus mykiss*)	24 *w.w.*	3*S*,3′*S*	[86]
	Arctic char (*Salvelinus alpinus*)	8.6 *w.w.*	—	[86]

^∗^ *d.w.*, dry weight; *w.w.*, wet weight.

**Table 3:** Second-order quenching rate constants for the quenching of singlet oxygen by astaxanthin.

Quenching rate constant (*k*_q_, 10^8^ M^–1^ s^–1^)	Method	Solvent	Ref.
5.9	Rose Bengal-sensitized photooxidation	DPP liposomes	[150]
0.19	Rose Bengal-sensitized photooxidation	Stearylamine and dimyristoylphosphatidylcholine liposomes	[151]
0.19	12-(1-Pyrene)-dodecanoic acid-sensitized photooxidation	Dimyristoylphosphatidylcholine liposomes	
140	Phenazine sensitization	Benzene	[152]
71.1	Thermal decomposition of 3-(1,4-epidioxy-4-methyl-1,4-dihydro-1-naphthyl) propionic acid as endoperoxide	Triton X-100 solution (5 wt%; 0.02 M phosphate buffer, pH 7.4)	[153]
118	Thermal decomposition of 3-(1,4-epidioxy-4-methyl-1,4-dihydro-1-naphthyl) propionic acid as endoperoxide	Ethanol:chloroform:D_2_O (50 : 50 : 1, *v*/*v*/*v*)	[154]
240	Thermodissociation of the endoperoxide of NDPO_2_	Ethanol/chloroform/H_2_O (50 : 50 : 1, *v*/*v*)	[155]
22	Thermodissociation of the endoperoxide 1,4-dimethyl-naphthalene	CDCl3	[156]
(18)		(CDCl_3_/CD_3_OD (2 : 1, *v*/*v*))	

**Table 4:** Antioxidant assays and methods for the determination of antioxidant activity for astaxanthin and its isomers.

Type	Method	Antioxidant activity	Unit	Reference
Astaxanthin	Free-radical scavenging activity (DPPH)	17.5 ± 3.6	*μ*g/mL	[165]
Astaxanthin		39.1 ± 1.14		[166]
All-*trans*		5.06	TE/mg	[164]
13-*cis*		6.49		
9-*cis*		8.85		
All-*trans*	Oxygen radical absorbing capacity for lipophilic compounds	7.65	TE/mg	
13-*cis*		13.22		
9-*cis*		11.16		
All-*trans*	Photochemiluminescence	92.22	*μ*mol TE/g	
13-*cis*		117.01		
9-*cis*		103.41		
Astaxanthin	Radical scavenging activity (ABTS)	7.7 ± 0.6	EC50, *μ*g/mL	[165]
Astaxanthin	*β*-Carotene bleaching activity	15.1 ± 1.9		
Astaxanthin	Singlet oxygen scavenging activity	9.2 ± 0.5		
Astaxanthin	Ferric reducing antioxidant power	0.5	mol *α*-TE/mol	[167]
Astaxanthin	ABTS bleaching assay (*α*TEAC)	0.8		
Astaxanthin	Luminol-chemiluminescence based Peroxyl radical scavenging capacity (LPSC)	26.3		

Note: ABTS: 2,2′-azino-bis-3-ethylbenzthiazoline-6-sulphonic acid; DPPH: 2,2-diphenyl-1-picrylhydrazyl; LPSC: luminal-chemiluminescence peroxyl radical scavenging; mol *α*-TE/mol: mol *α*-tocopherol equivalent per mol; TE: Trolox equivalent; *α*TEAC: *α*-tocopherol equivalent antioxidant capacity.
